# *In silico* analysis of drug resistance in wild type and mutant HIV-1 subtype d protease

**DOI:** 10.1186/1471-2334-14-S3-E23

**Published:** 2014-05-27

**Authors:** Punnagai Munusami, CS Vasavi

**Affiliations:** 1 Bioinformatics Division, School of Bio Sciences and Technology, Vellore Institute of Technology University, Vellore-632014, India

## Background

A general cause for therapeutic failure in people is due to the high resistance of HIV protease to antiretroviral drugs. The aim of the present work is focused on two objectives: to study the mutations responsible for the drug resistance using Stanford database and how the existing ART drugs are resistant to subtype d (wild type, major and minor mutations) using docking studies.

## Methods

The mutation frequency of subtype d in untreated persons was obtained from Stanford DR database, http://hivdb.stanford.edu/. Based on the database the wild type PTD sequence was generated. Crystal structure 3LZS showed more similarity based on BLASTp program. The protein structure 3LZS was used as a template to build wild type, major (L10V, N37D, K69Y), minor (K20I, L33I, P39T, Q61N), major+minor mutants of PTD using Modeller9v7. The docking studies of protease inhibitors (atazanavir, darunavir, indinavir, lopinavir, nelfinavir, ritonavir, saquinavir, tipranavir) with the wild type and mutant models were carried using AutoDock 4.2.5.

## Results

The docking studies revealed that contribution of high torsional energy in atazanavir affects the binding energy and leads to high level resistance to the wild type and the mutants. Indinavir showed susceptibility for the wild type PTD whereas nelfinavir showed susceptibility to all mutants and the susceptibility was due to less number of torsions and increased van der Waals, hydrogen bonding, desolvation and electrostatic energy contributions.

## Conclusion

The developed wild type and mutant model of PTD provides an insight to understand the effect of mutations and resistance towards the protease inhibitors.

